# Work-Related Asthma — 22 States, 2012

**Published:** 2015-04-10

**Authors:** Jacek M. Mazurek, Gretchen E. White

**Affiliations:** 1 Division of Respiratory Disease Studies, National Institute for Occupational Safety and Health, CDC

Work-related asthma* (WRA) is a preventable occupational disease associated with serious adverse health outcomes (1–3). Using the 2006–2009 Behavioral Risk Factor Surveillance System (BRFSS) Adult Asthma Call-back Survey (ACBS) data from 38 states and the District of Columbia, CDC estimated that among ever-employed adults with current asthma, the proportion of current asthma that is work-related was 9.0% (4). In 2011, the BRFSS cellular telephone samples were added to the traditional landline telephone samples and the weighting methodology was changed.† In 2012, a revised ACBS question on WRA diagnosis^§^ was asked. To provide updated estimates of current asthma prevalence and the proportion of asthma that is work-related, by state, CDC analyzed data from BRFSS and ACBS collected from 22 states using both landline and cellular telephone samples during 2012. This report summarizes the results of that analysis, which indicate that 9.0% of adults had current asthma and that among ever-employed adults with current asthma, the overall proportion of current asthma that is work-related was 15.7%. State-specific proportions of asthma that is work-related ranged from 9.0% to 23.1%. Distribution of the proportion of WRA significantly differed by age and was highest among persons aged 45–64 years (20.7%). These findings provide a new baseline after the implementation of changes in survey methodology (5) and the adoption of a revised WRA question. These results can assist states, other government agencies, health professionals, employers, workers, and worker representatives to better target intervention and prevention efforts to reduce the burden of WRA.

BRFSS is a state-based, random-digit–dialed telephone survey of the non-institutionalized U.S. civilian population aged ≥18 years that collects information on health risk factors, preventive health practices, and disease status.¶ The 2012 BRFSS included a standard set of core questions, 27 optional modules, and state-added questions. One of the optional modules, the CDC-funded ACBS, is designed to collect detailed information on asthma, including WRA. BRFSS respondents who answer “yes” to the question, “Have you ever been told by a doctor, nurse, or other health professional that you had asthma?” are invited to participate in ACBS.** Those who agree to participate are interviewed within 2 weeks of the BRFSS completion date. In 2012, ACBS was administered among adults in 22 states.

In 2011, in order to address the effect of an increasing number of cellular telephone–only households on BRFSS coverage, cellular telephone samples were added to landline telephone samples (5). To address this change and to reduce the potential for bias associated with declining response rates, BRFSS also adopted a new statistical weighting methodology (5). Also, in 2012, the content of the ACBS WRA section was revised. Adult data from 2012 BRFSS and ACBS collected from 22 states using both landline and cellular telephone samples are included in this analysis. The median response rate among the 22 states was 44.9% (range: 27.7%–56.8%) for BRFSS†† and 47.2% (range: 38.5%–60.6%) for ACBS.^§§^

For this analysis, BRFSS participants who responded “yes” to the questions, “Have you ever been told by a doctor or other health professional that you have asthma?” and “Do you still have asthma?” were identified as having current asthma. Ever-employed ACBS participants were those who indicated that they were currently employed full- or part-time or that they had ever been employed. Ever-employed adults with current asthma who responded “yes” to the question, “Have you ever been told by a doctor or other health professional that your asthma was caused by, or your symptoms made worse by, any job you ever had?” were classified as having WRA.

Data for 2012 from all 22 states collecting adult data using landline and cellular telephone samples were weighted¶¶ to account for noncoverage, unequal probability of sample selection, and nonresponse differences in the sample. Statistically significant differences in distribution were determined by using the Rao-Scott chi-square test of independence at p≤0.05.

In the 22 states, a sample of 205,755 adults participated in BRFSS (representing an estimated 137 million persons) and 9,893 adults participated in the ACBS (representing an estimated 18 million persons). In 2012, an estimated 9.0% of adults had current asthma in these 22 states (Table). The prevalence of current asthma significantly differed by age, sex, race/ethnicity, and education. Prevalence was highest among persons aged 45–64 years (9.4%), women (11.4%), blacks (12.5%), and those with less than a high school education (9.5%). By state, estimates of the current asthma prevalence ranged from 6.8% to 10.9%.

A total of 7,275 adults who participated in ACBS were ever-employed and had current asthma, representing an estimated 12 million adults in these 22 states. Of these, the estimated proportion who had WRA was 15.7% (an estimated 1.9 million persons) (Table). The proportion of WRA among ever-employed persons with current asthma differed significantly by age and was highest among persons aged 45–64 years (20.7%). By state, the estimated proportions of ever-employed adults with current asthma who had WRA ranged from 9.0% to 23.1%.

## Discussion

Among ever-employed adults with current asthma, 15.7% had WRA, indicating that an estimated 1.9 million WRA cases (new-onset and work-exacerbated asthma) could potentially have been prevented in these 22 states. These findings provide a new baseline to be compared with future estimates. Several factors need to be considered when interpreting these results. First, the 2012 data are not comparable methodologically with those collected during preceding years and should be used as a baseline to compare with subsequent survey results. The addition of cellular telephone–only households to the survey sample improved the representativeness of data collected by BRFSS and likely increased the coverage of respondents who are younger and who have a lower income, less education, an unmet need for medical care, and a higher number of risk factors for chronic diseases (5–8). In 2012, the estimated median proportion of cellular telephone-only households in the 22 states included in this study was 36.7% (range: 23.5%–49.4%).*** Moreover, weights used in this analysis were computed by using an iterative proportional fitting (i.e., “raking”) method, which offers several advantages over the method used previously (i.e., “poststratification”). Raking allows for the introduction of more demographic variables and the incorporation of telephone ownership into statistical weighting, thus reducing the potential for bias and improving the representativeness of estimates (5,8). Finally, in 2012 a revised question that identifies respondents with WRA was asked as part of ACBS.

Administration of ACBS should continue to allow state asthma programs to monitor the proportion of asthma that is work-related. In addition, the National Institute for Occupational Safety and Health (NIOSH) supported an optional module in 2013 and 2014††† to collect information on the current industry and occupation of participants. These data will inform the development of public health intervention strategies (i.e., occupations suspected to place workers at high risk for development of WRA should be evaluated, and effective exposure control measures should be implemented to prevent WRA) (4). Because a WRA diagnosis offers unique opportunities for prevention for the patient and among workers with similar occupational exposures, health-care providers should ask workers with asthma about occupational exposures and be alert to potential associations between workplace exposures and asthma symptoms (2).

The findings in this report are subject to at least six limitations. First, measures of current asthma and WRA were based on self-report and not validated by medical records review or follow-up with health-care providers. Previous studies have found self-report of adult asthma to be reliable compared with reviews of medical records (9). Moreover, because of the potential impact of a work-related asthma diagnosis on a patient’s work (3), it is likely that respondents would report their work-related asthma history accurately whereas a diagnosis that did not lead to changes at work might be forgotten. Second, a study showed that clinicians documented occupational exposures in only 7% of adult-onset asthma cases (10) indicating that WRA is underdiagnosed in the United States; thus results are likely underestimates of the true proportion of WRA. Third, no data were available in BRFSS to assess the prevalence of current asthma among ever-employed adults. Therefore findings on the prevalence of current asthma and the proportion of current asthma that is work-related were calculated using different populations and should be interpreted with caution. Fourth, the data used in this analysis are limited to adults living in 22 states participating in ACBS; therefore, the estimates are not nationally representative or representative of nonparticipating states. Fifth, because the BRFSS and ACBS median response rates were <50%, nonresponse bias might have affected the results. Finally, small sample sizes for some subpopulations resulted in estimates with wide confidence intervals. Additional years of data are needed to calculate more precise estimates.

For many states, ACBS provides the only state-based estimates of WRA. These new, improved results can assist states, other government agencies, health professionals, employers, workers, and worker representatives to prioritize disease intervention and prevention efforts to reduce the burden of WRA.

What is already known on this topic?

Work-related asthma (WRA) is a preventable, often underdiagnosed, occupational lung disease. On the basis of the 2006–2009 Behavioral Risk Factor Surveillance System Adult Asthma Call-back Survey (ACBS) data from 38 states and the District of Columbia among ever-employed adults with current asthma, the overall proportion of current asthma that is work-related was estimated to be 9.0%.

What is added by this report?

An estimated 1.9 million cases of asthma among adults were work-related (new-onset and work-exacerbated), accounting for 15.7% of current asthma cases among ever-employed adults, and thus could potentially have been prevented in the 22 states conducting ACBS in 2012. This estimate provides a new baseline for comparison with future estimates and reflects Behavioral Risk Factor Surveillance System methodology changes including new, improved statistical weighting, improved data collection by addition of cellular telephone samples to landline telephone samples, and revision of the ACBS question on WRA diagnosis to specifically ask about asthma caused by or made worse by work.

What are the implications for public health practice?

For many states, ACBS provides the only state-based estimates of WRA. These new results can assist states, other government agencies, health professionals, employers, workers, and worker representatives in prioritizing disease intervention and prevention efforts to reduce the burden of WRA.

## Figures and Tables

**TABLE:** Prevalence of current asthma* in adults and proportion of ever-employed† adults with current asthma who have been told by a health professional that their asthma was work-related,§ by state and selected characteristics — Behavioral Risk Factor Surveillance System (BRFSS) and Adult Asthma Call-Back Survey (ACBS), 22 states, 2012

Characteristic	Adults				Ever-employed adults with current asthma			
	
	No. in sample¶	Weighted no. (in thousands)**	Prevalence of current asthma		No. in sample¶	Weighted no. (in thousands)**	Proportion with work-related asthma	
	
			%**	(95% CI)			%**	(95% CI)
**Total**	**205,755**	**137,831**	**9.0**	**(8.7–9.2)**	**7,275**	**12,270**	**15.7**	**(13.7–17.7)**
**Age group (yrs)** †† **,** §§								
18–44	57,172	65,456	8.8	(8.4–9.2)	1,514	5,562	13.0	(10.0–16.1)
45–64	79,883	46,997	9.4	(9.0–9.8)	3,363	4,550	20.7	(17.2–24.1)
≥65	66,978	24,566	8.7	(8.2–9.1)	2,373	2,133	12.1	(9.3–15.0)
**Sex** ††								
Male	84,488	67,117	6.4	(6.1–6.7)	2,122	4,275	17.6	(13.5–21.6)
Female	121,267	70,714	11.4	(11.0–11.8)	5,153	7,995	14.8	(12.6–16.9)
**Race/Ethnicity** †† **,** ¶¶								
White	158,929	86,226	9.2	(8.9–9.4)	5,729	8,430	14.9	(13.1–16.7)
Black	12,899	12,829	12.5	(11.4–13.5)	554	1,299	12.3	(7.2–17.4)
Hispanic	15,907	24,813	6.8	(6.1–7.4)	332	1,452	18.2	(10.0–26.4)
Other race	15,498	12,407	8.7	(7.7–9.7)	583	993	23.5	(10.7–36.2)
**Education** ††								
<High school	79,948	60,017	9.5	(9.1–9.9)	2,686	4,574	16.1	(13.1–19.0)
≥High school	125,115	77,297	8.6	(8.3–8.9)	4,584	7,694	15.5	(12.9–18.2)
**State**								
California	14,574	28,845	8.8	(8.2–9.5)	355	2,744	14.2	(8.5–19.9)
Hawaii	7,582	1,080	8.9	(7.9–9.9)	228	92	9.0	(3.8–14.2)
Illinois	5,579	9,810	8.5	(7.4–9.6)	215	729	16.0	(8.3–23.7)
Indiana	8,645	4,946	9.1	(8.3–9.8)	330	447	16.2	(10.9–21.4)
Iowa	7,166	2,345	8.1	(7.2–8.9)	233	181	18.0	(12.1–23.8)
Michigan	10,499	7,583	10.5	(9.6–11.3)	546	836	14.7	(10.3–19.1)
Mississippi	7,788	2,236	8.1	(7.3–9.0)	310	191	20.6	(13.7–27.5)
Missouri	6,754	4,609	10.4	(9.3–11.5)	278	449	23.1	(15.0–31.3)
Montana	8,679	781	9.5	(8.6–10.3)	292	75	14.5	(9.0–20.0)
Nebraska	19,173	1,391	7.4	(6.9–7.9)	633	101	15.7	(11.8–19.6)
Nevada	4,846	2,078	7.4	(6.3–8.4)	159	161	13.7	(6.6–20.8)
New Hampshire	7,530	1,041	10.2	(9.2–11.3)	294	109	14.4	(7.8–20.9)
New Mexico	8,776	1,582	9.2	(8.5–10.0)	375	155	13.5	(8.6–18.4)
New York	6,060	15,274	9.3	(8.3–10.3)	190	1,332	13.6	(6.0–21.2)
Ohio	13,026	8,856	10.5	(9.7–11.2)	424	948	20.3	(12.3–28.3)
Oklahoma	8,015	2,886	10.2	(9.3–11.0)	249	286	13.9	(7.2–20.6)
Oregon	5,302	3,039	10.6	(9.5–11.8)	218	315	—***	—
Pennsylvania	19,958	10,025	10.1	(9.4–10.8)	696	898	14.6	(10.9–18.5)
Texas	9,129	19,185	6.8	(6.1–7.6)	245	1,257	17.6	(10.2–25.0)
Vermont	6,056	501	10.9	(9.8–12.0)	271	57	14.3	(7.6–21.1)
Washington	15,319	5,336	9.7	(9.1–10.3)	515	515	14.2	(9.9–18.5)
Wisconsin	5,299	4,402	8.6	(7.4–9.7)	219	394	21.1	(13.4–28.9)

**Abbreviation:** CI = confidence interval.

* Based on a “yes” response to both questions, “Has a doctor, nurse, or other health professional ever told you that you had asthma?” (BRFSS) and “Do you still have asthma?”

† Current employment status described as “employed full-time” or “employed part-time” or a “yes” response to the question, “Have you ever been employed?”

§ Based on a “yes” response to the question, “Have you ever been told by a doctor or other health professional that your asthma was caused by, or your symptoms made worse by, any job you ever had?”

¶ Landline and cellular telephone combined unweighted sample size.

** Weighted to the state population using the survey sample weights for each BRFSS and ACBS participant.

†† For current asthma: Rao-Scott chi-square test; p-value <0.01.

§§ For work-related asthma: Rao-Scott chi-square test; p-value <0.01.

¶¶ Persons identified as Hispanic might be of any race. Persons identified as white, black, or other race are all non-Hispanic.

*** Relative standard error >0.30; estimate suppressed.

## Footnotes

* WRA includes occupational asthma (i.e., new-onset asthma caused by factors related to work) and work-exacerbated asthma (i.e., preexisting or concurrent asthma worsened by factors related to work).

† Additional information is available at http://www.cdc.gov/brfss/annual_data/2012/pdf/Overview_2012.pdf and at http://www.cdc.gov/brfss/acbs/2012/pdf/ACBS_2012.pdf.

¶ Additional information and survey data and documentation available at http://www.cdc.gov/brfss/about/index.htm and at http://www.cdc.gov/brfss/annual_data/annual_data.htm#2013.

** Additional information and survey data and documentation available at http://www.cdc.gov/brfss/acbs/index.htm.

†† Source: CDC. Behavioral Risk Factor Surveillance System, 2012 Summary Data Quality Report, July 3, 2013. Available at http://www.cdc.gov/brfss/annual_data/2012/pdf/summarydataqualityreport2012_20130712.pdf.

¶¶ CDC. The BRFSS Data User Guide, August 15, 2013. Available at http://www.cdc.gov/brfss/data_documentation/PDF/UserguideJune2013.pdf.

*** Source: Blumberg SJ, Ganesh N, Luke JV, Gonzales G. Wireless substitution: state-level estimates from the National Health Interview Survey, 2012. Natl Health Stat Report 2013;1–16. Available at http://www.cdc.gov/nchs/data/nhsr/nhsr070.pdf.

††† NIOSH will also support the Industry and Occupation optional module in 2015 and 2016.
